# A Conceptual Framework for User Trust in AI Biosensors: Integrating Cognition, Context, and Contrast

**DOI:** 10.3390/s25154766

**Published:** 2025-08-02

**Authors:** Andrew Prahl

**Affiliations:** Wee Kim Wee School of Communication and Information, Nanyang Technological University, Singapore 637718, Singapore

**Keywords:** artificial intelligence, trust, human–machine interaction

## Abstract

Artificial intelligence (AI) techniques have propelled biomedical sensors beyond measuring physiological markers to interpreting subjective states like stress, pain, or emotions. Despite these technological advances, user trust is not guaranteed and is inadequately addressed in extant research. This review proposes the Cognition–Context–Contrast (CCC) conceptual framework to explain the trust and acceptance of AI-enabled sensors. First, we map cognition, comprising the expectations and stereotypes that humans have about machines. Second, we integrate task context by situating sensor applications along an intellective-to-judgmental continuum and showing how demonstrability predicts tolerance for sensor uncertainty and/or errors. Third, we analyze contrast effects that arise when automated sensing displaces familiar human routines, heightening scrutiny and accelerating rejection if roll-out is abrupt. We then derive practical implications such as enhancing interpretability, tailoring data presentations to task demonstrability, and implementing transitional introduction phases. The framework offers researchers, engineers, and clinicians a structured conceptual framework for designing and implementing the next generation of AI biosensors.

## 1. Introduction

Sensors are everywhere. They measure athlete reaction times on a volleyball court [1], detect gait freezing in Parkinson’s disease [2], and even track micro air-pollution particulates in congested urban centers [3]. They safeguard structural health in civil infrastructure and monitor the real-time performance of lithium battery modules [4,5]. In biomedical domains, they promise countless possibilities ranging from continuous blood-oxygen surveillance to real-time detection of arrhythmias, respiratory distress, or neural signals [6,7,8,9,10]. Driven by advances in sensing and artificial intelligence, in the domain of medicine and wellness sensors are increasingly encroaching on the “sensitive” (and often subjective) facets of human experience.

Trends in recent biomedical sensors mirror their increasing application to more intangible areas. Traditional hardware-oriented breakthroughs (e.g., improved optical or fiber-optic techniques) now feature alongside data-driven methods such as machine learning, deep learning, or hybrid approaches [11,12]. While the result is sharper accuracy, faster responses, and broader deployment, user perceptions are not always on board. A new ECG patch might be clinically impressive, but do individuals truly trust it to interpret personal data like stress or pain level? Even developers concede that technical brilliance alone will not assure user trust. Indeed, a market analysis argues that lack of user trust is now a primary brake on wearable adoption and adherence [13,14]. Furthermore, a recent pregnancy-monitoring review concludes, “there are still several challenges that need to be addressed, such as public trust, personal data security, and the risk of AI error… Building public trust in novel areas is a difficult and lengthy process” [11] (p. 6426). The first step to “public” trust is user trust. These concerns surface in almost every new application of biosensors, yet the field still lacks a systematic playbook for resolving them.

Such challenges intensify when sensors integrate artificial intelligence (AI) in measurement and/or decoding. The classic signal measurement scheme is no longer an end, now algorithms are increasingly providing diagnoses, predicting events or sensations, or merging sensor data for insights that feel far more interpretive than before. For example, recent research demonstrated the classification of knee subchondral sclerosis severity through AI-driven analysis of radiographic images [15]. Here, a deep learning model moved beyond simple data gathering by diagnosing subchondral sclerosis grades with sensitivity and specificity that rivaled expert human readers. It did not merely acquire images from a radiographic sensor; it analyzed those images, derived insights, and arrived at a sophisticated classification outcome. Such an expanded role for sensors is exciting but delicate. For example, how do people—or their physician—respond when an AI sensor system contradicts their own self-assessment (e.g., “I feel fine but the machine says I am unwell”)?

The complicated issue of user and practitioner trust is growing in significance as AI advances. We therefore present a conceptual framework, Cognition, Context, and Contrast (CCC), to understand how and why certain sensor-based or AI-powered solutions are embraced versus distrusted. We review research in related domains on human expectations of technology, the domain of measurement (intellective versus judgmental), and the role of prior experiences. The study has three specific goals: (i) synthesize scattered findings in related domains into one coherent framework, (ii) show how this framework can improve future research and design of biosensors, and (iii) suggest resulting practical steps that engineers, clinicians, and administrators can operationalize in the real-world today and going forward. Because sensor technologies evolve faster than large-scale field trials can keep pace, new conceptual frameworks are useful; recent examples include street-level cyber-physical models [16] and blockchain-IoT fish-supply frameworks [17]. Our CCC framework serves the same forward-looking role. Our overarching aim is to clarify how sensor credibility takes shape today and, going forward, into the era of increasingly AI-powered sensors.

## 2. Understanding Sensor Trust: A Conceptual Gap

Despite impressive strides ranging from sensor-based athletic monitoring to advanced wearable devices, research often overlooks the tricky aspects of subjective measurement. For example, acceptance of intangible indicators (e.g., pain severity, fatigue, stress level) differs from acceptance of more straightforward, intellective data (e.g., heart rate, body temperature). Moreover, while AI is transforming data interpretation, we still lack a concise conceptual framework to explain how AI features may provoke new biases or stereotypes that shape trust. Without a conceptual framework that accounts for user perception, the latest AI-sensor solutions risk confusion or outright rejection in critical settings like orthopedics or neurology.

This gap motivates the CCC model. We argue that bridging AI sensor technology with user trust involves understanding (1) *cognition*: the expectations and mental models users already hold about sensors and AI, (2) *context*: the nature of the measured phenomenon, and (3) *contrast*: if the sensor replaces or complements existing sources of information. Below, we detail the CCC model and highlight how it applies to sensor use cases, including those that reach beyond objective measurements and tread into more personal domains. Furthermore, we show how AI’s increasing use in sensors affects each consideration. Our overarching study aim is to clarify the conditions under which AI-enhanced sensors can earn user trust and fulfill their potential in both clinical and non-clinical spheres.

### Positioning CCC in the Trust-in-Automation Landscape

The CCC framework sits at the intersection of three influential research streams. First, theoretical work on trust in automation highlights dispositional, situational, and learned factors that shape reliance over time [18,19,20]. CCC adopts the same dynamic spirit but adds granularity on what people are evaluating. By unbundling cognition, context, and contrast, CCC specifies which situational cues matter most when sensors migrate from benign step-counters to diagnostic advisors.

Second, acceptance models such as the Unified Theory of the Acceptance and Use of Technology (UTAUT) and Technology Acceptance Model (TAM) emphasize perceived usefulness, ease of use, and social influence [21,22]. These macro-level predictors remain relevant, yet evidence shows that once a device moves into safety-critical territory, trust antecedents can eclipse usability [19,23]. CCC therefore complements UTAUT and similar models by foregrounding two constructs absent in general adoption models: (i) demonstrability—the clarity with which a “correct” answer can be shown; and (ii) contrast explains the psychological shock that arises when an algorithm supplants a familiar human workflow.

Third, “algorithm-aversion” and “machine-heuristic” research documents people’s tendency to over-trust apparently objective systems until the first salient failure, after which trust plunges [20,24,25]. CCC explains why this swing is so pronounced: inflated initial confidence flows from the cognition stereotype of machine infallibility, while the steep drop is amplified by contrast when a dependable human benchmark is suddenly absent.

In short, CCC does not replace existing theories; it integrates their most predictive elements and adds two missing pieces—task demonstrability and contrast effects—providing the first framework to link measurement ambiguity and human displacement to user trust in biosensing. These additions turn broad adoption factors into a useful lens for designers who must decide when to simplify design, how to roll-out new technologies, and when to temper perfection stereotypes.

## 3. Cognition: Stereotypes and Trust in Humans Versus Machines

An enduring question in human–technology interaction is the extent to which individuals trust a non-human, especially for tasks which have real consequences [18]. Historically, trust research distinguished between interpersonal trust among humans and trust in automation or machine aids [20,25,26]. Here, we adapt these ideas to focus on biomedical sensors, recognizing that sensors do more than merely gather data: many now integrate artificial intelligence or advanced signal processing that effectively advises clinicians and patients in high-stakes decisions (e.g., diagnosing irregular heartbeats or detecting pain episodes [27,28]). Cognition is defined as the mental models, expectations, and prior beliefs users hold about machine agents. Operationally, cognition can be captured via instruments like the Machine-Heuristic Scale [29] and Perfect-Automation Schema Measurement [30].

A recurring stereotype about machines is that they provide neutral, data-driven outputs but lack the intuitiveness, empathy, or context sensitivity that human experts bring [31]; see [32] for an early discussion in computer-based expert judgements. Qualitative work with AI pain-apps for dementia care illustrates the point: carers frequently mistrusted the algorithm’s pain score until a clinician demonstrated concordance with observable discomfort [33]. In biomedical contexts, such as wearable ECG monitors, the assumption may be that sensors deliver purely objective insights. Patients and clinicians often welcome this objectivity, expecting sensor data to be untainted by subjective biases. Consequently, sensors can experience a positivity bias [20,34,35,36,37], whereby users assume near-perfect measurement capabilities until proven otherwise.

Simultaneously, people may exhibit reservations about relying on a device that lacks human qualities like compassion. When a patient feels pain, but the sensor indicates “no abnormal measurement” (e.g., skin conductance remains normal, or pupillometry suggests lower arousal), the mismatch can trigger distrust or skepticism. This tension mirrors earlier research showing that humans often resent being treated as “mere numbers” by automated decision aids [23], (p. 581). While sensors can bolster objectivity, there is also the widespread belief that machines oversimplify complex states and cannot genuinely understand or care [25,29,38].

### Expectation of Superior Performance and Quick Distrust

Numerous studies on human–automation trust highlight how users initially overestimate the reliability of computerized systems [20,39]. In healthcare, individuals similarly assume a biomedical sensor—especially one leveraging sophisticated algorithms—must be more precise than any single human observer [29,32]. Age and device form-factor also impact these mental models: a 1158-respondent UTAUT study showed that trust translated into usage intention far more steeply for a contact-less radar sensor than for a familiar wearable—and the slope was steeper still among older adults [40].

Yet, paradoxically, once a sensor fails visibly (like missing a known arrhythmia or incorrectly flagging a false alarm), trust declines quickly. As prior work on automation bias indicates, human operators tend to be highly attentive to machines that deviate from the perfect automation stereotype [20,41,42]. In biomedical scenarios, misses on “obvious” events (e.g., a patient’s pain flare-up is not captured or the sensor incorrectly shows normal glucose when the patient is symptomatic) may provoke stronger distrust than would a human oversight—because people generally expect human fallibility but hold sensors to a stricter accuracy standard [20].

A parallel distinction also emerges in how users explain or justify errors from sensors vs. humans. In interpersonal contexts, if a healthcare provider misdiagnoses, people might invoke ideas of limited time, the complexity of a case, or empathic but misguided reasoning [43]. In contrast, users perceive sensors as invariant, that is, a sensor’s coding is fixed, so if it errs, it must be systematically untrustworthy and a sensor cannot “self-correct” the way a human could [20]. As an illustrative example, consider an AI-powered wearable sensor designed to detect stress [44]. Such a sensor might be considered exceptional in capturing subtle physiological cues. Yet if it recommends a response or “interpretation” that feels off to the user—perhaps dismissing a stressful event as “normal”—the user may abruptly lose trust, attributing the discrepancy to the sensor’s inability to truly “feel” stress. Under such conditions, people will sometimes revert to self- or other human-based interpretation and the sensor’s potential may not be realized. For example, recent research on wellness wearables included interviews with adult users of fitness, sleep-tracking, and diet apps. Trust hinged on perceived accuracy: a single implausible reading (e.g., 3 h of sleep after an actual full night’s rest) could prompt abandonment of the device, whereas consistently credible feedback motivated deeper engagement and additional metric tracking [45]. The same phenomenon can occur even in professional medical settings, where unexpected false alarms can erode confidence more rapidly than multiple small errors from a human nurse. Furthermore, each comparison with a human benchmark (described later in the Section 5) feeds straight back into expectations, sometimes inflating the effects.

In summary, the stereotypes about sensors in biomedical contexts (i.e., that they are perfectly objective yet devoid of empathy, or that they deliver flawless data unless they catastrophically err) parallel long-standing views in automation trust research [32,39]. Recognizing that people may initially over-trust sensor readings, yet become hyper-critical upon any error, is crucial to fostering balanced, long-term trust of such tools [18]. Ultimately, these cognitive factors rooted in persistent stereotypes of “machine vs. human” underscore the need for thoughtful sensor design and user education. By clarifying limitations, providing transparent feedback on how sensor algorithms work, and offering guidance when interpretive nuances arise, biomedical sensors can earn the optimal degree of trust—rather than fall victim to the “all or nothing” cycle that often plagues machine-based advisement.

## 4. Context: Task Demonstrability and Its Influence on Trust of Biomedical Sensors

A continuum of decision-making tasks anchored by intellective and judgmental ends has long been proposed to understand how individuals differentially rely on various forms of advice or assistance [46]. Although originally employed in group decision-making studies, this intellective-judgmental continuum has become a useful model in advice and technology research more broadly [47]. Under this framework, we can conceptualize high-demonstrability (or “intellective”) tasks as decisions with demonstrably correct answers. For example, an algebra problem has a correct answer and any advice provided to a decision-maker suggesting that the correct answer is demonstrably correct or incorrect—assuming the advice receiver shares the same conceptual system with the advisor. Conversely, judgmental tasks involve uncertain future states or personal opinions; there is no single demonstrably correct piece of advice. Examples include deciding whether an artistic performance warrants an award or what level of discomfort in a treatment scenario is acceptable, given risk–benefit tradeoffs.”

Shifting to a biomedical context, Context refers to the task environment in which a sensor operates, characterized by (i) demonstrability (clear vs. ambiguous ground truth), (ii) ethical or moral stakes, and (iii) perceived consequence of error. Context can be measured by demonstrability indices adapted from Laughlin and Ellis [46] and risk inventories [48]. The consideration of task demonstrability illuminates why certain biomedical sensor use cases appear straightforward (“Does the sensor reliably detect bradycardia?”) while others are more ambiguous (“How should we weigh a sensor’s streaming data about pain intensity when the patient’s self-report differs?”). For instance, the difference between a pulse oximetry device that simply flags oxygen saturation below 92% (a high-demonstrability threshold) and an AI-powered wearable that “interprets” the meaning of chronic pain signals from electromyography (a more subjective, low-demonstrability domain) can be stark. Field studies of multi-device sleep-tracking show why. In a two-week study, nightly sleep duration differed across four commercially validated trackers and users’ own perceptions of sleep quality; interviewees said such discrepancies ‘made it hard to know which reading to believe,’ eroding confidence in the entire class of devices [49]. On the other hand, in a survey of over 400 people with diabetes, 80% expressed moderate-to-great trust in AI-driven glucose monitors—evidence that high-demonstrability metrics (blood sugar) quickly earn credibility [50].

### 4.1. Moral and Ethical Overlays

Complicating the intellective-judgmental spectrum for biosensors, medical decisions can have moral or ethical ramifications. Obviously, such decisions are lower in demonstrability because objective “right answers” are less clear. For example, recalling the overstretched resources in some hospitals during the COVID-19 pandemic, wearable sensors may precisely monitor real-time lung function, but deciding who receives specialized treatments first may hinge on personal or institutional values. Such moral decisions become “judgmental” because they require subjective weighting of individual and collective patient safety and patient autonomy—things that are not suited to numerical description [51,52]. Thus, we see how context determines how clinicians, patients, and laypersons adopt or question sensor outputs. Automated or algorithmic “advice,” even if highly quantitative, does not always resolve the deeper, more value-laden facets of a decision [52]. This can inspire human skepticism of purely quantitative guidance, mirroring the historical debate in clinical vs. actuarial judgments [53] in medicine.

Privacy concerns can also interact with demonstrability, a recent field study including a range of demonstrability, employees asked to share objective fitness metrics (steps, heart-rate) accepted monitoring far more readily than when the same wearable reported inferred stress scores—data perceived as subjective, less verifiable, and ethically intrusive [54]. Perhaps subjective decisions are perceived as more risky, which would be an additional contextual moderator. For example, in a diagnosis task experiment, results showed that when potential harm was high, users with automated decision support verified the system’s advice significantly more often, exhibiting restrained behavioral trust compared with the same tool in a low-risk scenario [55].

### 4.2. Implications for Trust and Sensor Acceptance

**Figure 1:**
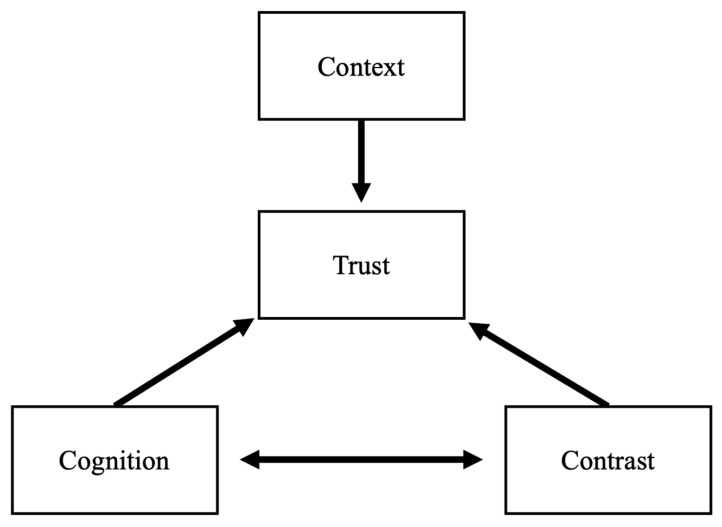
The CCC Model: Task Context sets a baseline level of trust. User Cognition and Contrast both act on Trust directly and affect each other.

Considering the demonstrability of the measurement task or sensor output is important for system designers. Context sits above the model because its demonstrability and ethical stakes can raise or depress trust directly, even before users consider sensor outputs (Figure 1). High-demonstrability tasks align well with sensors that can produce clear, quantifiable, and checkable metrics. Such tasks may exaggerate the expectation of the perfect machines discussed earlier. On the other hand, in tasks where moral, emotional, or deeply subjective judgments are in play, individuals may be skeptical of purely quantitative sensor outputs.

**Figure 2:**
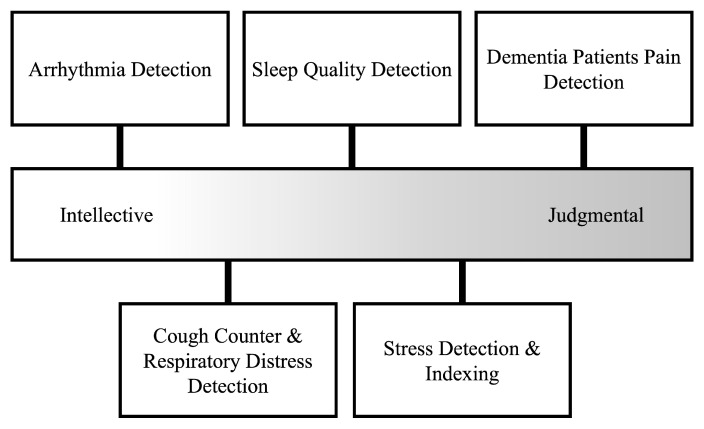
Intellective tasks (**right**) have clear ground truth (e.g., arrhythmia beats per hour [10]). Judgmental tasks (**left**) rely on subjective interpretation (e.g., AI pain scores in non-verbal dementia [27,33]). Mid-band examples blend both—sleep-tracker consensus still needs user appraisal [49], while cough-rate or stress-index sensors [9,56] combine objective signals with situational user feelings. Positioning of sensor technologies is for illustration only; each sensor and use-case will exist in a unique intellective–judgmental environment.

In practical terms, sensor designers and healthcare institutions could consider tailoring the presentation of sensor outputs, offering clear quantitative data for high-demonstrability tasks but ensuring more interpretative or empathic guidance for judgmental contexts (e.g., “Your [wearable sensor] indicates stress, how do you feel right now?”) (Figure 2). Such measures can temper the heuristics that might otherwise reject or blindly accept sensor data. It is thus critical for system designers to understand that as decisions shift toward moral or subjective domains, trust hinges on whether the device is seen as capable of handling human values. And, if users’ cognitive expectations amplify or reduce the perceived mismatch between the objective, “perfect” machine and the depth of personal judgment required.

## 5. Contrast: Effects of Shifting to Sensor-Based Systems

Recent analyses predict that AI or other forms of automation will increasingly replace human labor in various sectors over the coming decades [57,58,59,60]. Popular discussions of this trend often highlight the dire implications for workers who lose their jobs. However, far less attention is devoted to how the remaining humans—those who must now rely on machines—emotionally and behaviorally respond when a beloved colleague or trusted advisor is replaced by automation. Contrast captures the comparison frame users invoke when a sensor replaces, augments, or introduces capabilities relative to existing human or technological practices. It may be measured through perceived-replacement items that can be customized to any situation (e.g., “This device replaces a task nurses used to do”; “This sensor provides new information that was previously unavailable”).

In biomedical contexts, the contrast phenomenon most clearly plays out when a sensor-based system takes over key tasks previously performed by a human. For instance, automatically tracking a patient’s heart rhythm instead of relying on a technician’s periodic ECG checks, or using AI-driven image analysis in place of a radiologist’s initial screening. Such substitutions can trigger contrast effects, wherein the perceived differences between the replaced human approach and the newly introduced sensor are especially large. For example, a recent case study of fall-detection pendants found many seniors explicitly preferred a cane or human aide, judging the automated alert as uncaring and unhelpful compared to human watchers [61].

Contrast effects are well-documented in perception and decision-making research. They occur when exposure to one target of evaluation (such as a longstanding human-based assessment) influences how people subsequently evaluate another target (like a novel sensor or AI adviser) [62] In fields such as organizational psychology, it has been shown that comparing one co-worker’s performance to another’s can alter how an observer perceives each person’s strengths and weaknesses [63]. By the same logic, once a hospital staff or patient has grown accustomed to human-based measurement, the abrupt replacement by an autonomous wearable sensor can intensify perceptions, good or bad, of the new technology. Those intensified perceptions can, in turn, affect expectations carried into the next encounter.

The increased intensity of perception applies to the expectations that people have about machines (reviewed in the Section 3 above). Because contrast effects heighten people’s awareness of differences, missteps by the new system often lead to exaggerated trust declines (beyond the decline if the system had not replaced a human). It is important, therefore, for system developers, organizational leaders, and patient-facing physicians to remember that AI comes with the presumption of invariance and correctness [20,38]. This discrepancy grows more intense if the sensor explicitly replaced a conscientious human professional.

While the consideration of contrast effects may seem exclusive to situations that sensors replace a prior source of data, it is also important to consider the effects of cutting-edge AI-driven biosensors that provide insights which were never available before, such as predicting well-being, or the “sense of contentment, happiness, and fulfillment in life” [56] or predicting defecation events [64]. These capabilities may appear magical, which, on the positive side, can excite potential users. Essentially, contrast effects are muted, or even absent, when a sensor delivers a capability no human previously offered. For example, a novel PEC biosensor for the detection of lung cancer offered rapid detection unavailable before, so clinicians are likely to view it as an additive breakthrough rather than a rival [65]. For patients, there can be a negative side in causing wariness: individuals might doubt that this new technology is fully understood by their physicians. In practice, to manage the contrasting perceptions of enthusiasm vs. incredulity, developers might consider highlighting the system’s validated accuracy as well as placing disclaimers on the scope of AI’s interpretation.

From a practical standpoint, anticipating contrast effects is key to integrating new biomedical sensors successfully. Training sessions, transparent communication of each system’s capabilities, and transitional overlaps—where both human-led measures and sensor data are briefly used in parallel—may temper extreme comparisons. Likewise, clearly outlining what the sensor does (e.g., offering unique data streams previously unavailable) can help anchor user perceptions of the device in its strengths, rather than the frame of the device as a wholesale replacement.

## 6. CCC Operationalized: Practical Steps for Improving Sensor Acceptance

The value of the Cognition-Context-Contrast framework lies in how precisely it can map specific actions to specific trust problems. Below, each dimension is re-examined through the lens of recent sensor case studies to show what must happen—at design desk, bedside, and boardroom—before AI-enabled wearables move from prototype to routine care.

Cognition: Trust begins with what users think the system is. Device engineers therefore need to ship every product with an eye to interpretability. Explanations should be standard. These design features can decrease the perfection stereotype before it forms and give users and physicians a more straightforward understanding or narrative about how the system works and potential flaws. Controlled studies show that briefing users on a system’s limits moderates over-reliance and cushions trust erosion after errors. A recent human-autonomy teaming experiment found that teams receiving “trust calibration” training maintained more appropriate reliance despite agent failures [66]. Additionally, demographic-tailored explanations can be considered: studies show that older adults require a steeper trust-curve than digital natives when confronting unfamiliar form-factors such as radar sensors [40].

Context: The demonstrability of a measurement suggests how its output should be framed. In intellective domains (e.g., blood-pressure) a yes/no signal at an absolute threshold suffices. In judgmental domains, the same numeric certainty risks alienating users. Client-facing physicians should adopt contextual language. For example, alerts on fetal risks derived from sensors [14] become less confrontational when introduced with emotional considerations instead of categorical decisions. Administrators and industry leaders have a role to play as they can encourage policies such that—during patient consultations—any output tied to quality-of-life or sensitive topics should not be presented only as context-absent data.

Contrast: Adoption falters most violently when an AI sensor displaces a familiar routine overnight. Some current UK deployment guidance in radiology therefore urges a “shadow-mode” phase—running new technology silently while clinicians continue to rely on legacy thresholds—so staff can compare outputs before the cut-over [67]. Similar dual-tracking should be written into procurement policy. For example, a glucose predictor that replaces a beep-based monitor must include a fall back options to prior methods during the first month(s). Designers or organizational leaders can ease the hand-off further by offering periodic reports of how new technology compares to old methods.

While the CCC levers operate at the level of individual clinician-sensor interactions, they also generalize to enterprise scale. First, hospitals can institutionalize Cognition interventions via mandatory “trust-calibration” e-learning modules coupled with phased credentialing; every new hire completes the module before being granted write access to sensor dashboards. Second, Context can be operationalized in electronic-health-record middleware: task-type metadata (high-demonstrability vital vs. low-demonstrability pain score) flow through an API so the user interface automatically switches between binary alerts and richer confidence bands, ensuring consistent presentation across thousands of workstations. Third, to manage Contrast at scale, IT governance boards can require a two-to-four-week “shadow-mode” period—new AI outputs are logged but not acted upon—whenever a sensor displaces a legacy workflow; a trust-metrics dashboard then displays false-alarm rates, override counts, and clinician comments system-wide so leadership can spot hot-spots before full cut-over. Because these controls are policy-driven rather than device-specific, the same playbook works for a single ward piloting an optical biosensor or a multi-hospital network rolling out AI imaging triage, thereby demonstrating CCC’s scalability across diverse user populations and widescale AI adoption.

These measures translate CCC from abstract lens to an operational checklist. In the cumulative evidence now appearing in Sensors—from assessing pain in dementia patients [27] to weight-estimation insoles [68]—each intervention has begun to show measurable gains in sustained use. The implication is straightforward: with CCC-aligned engineering, bedside communication, and institutional policy, AI biosensors can move beyond proof-of-concept and into dependable, everyday practice.

## 7. Limitations, Boundary Conditions, and Validation

The CCC framework is bounded in several ways. Conceptual scope is the first limitation: CCC addresses antecedents of trust and calibrated reliance, not the full spectrum of normative concerns (e.g., privacy, informed consent, data governance). Designers must therefore pair CCC insights with dedicated ethical guidelines and regulatory standards. Second, cultural and generational differences can moderate each dimension [19,69]. Collectivist cultures, for instance, often place higher deference on expert authority and may show greater baseline faith in medical technologies endorsed by institutions, yet simultaneously exhibit stronger privacy concerns when data move beyond the bedside. High-power-distance contexts may soften contrast effects (e.g., users are accustomed to hierarchical expertise shifts) whereas individualistic settings might intensify skepticism when a known clinician is replaced by an opaque algorithm [69]. Younger users may demonstrate faster trust recovery after algorithmic errors than older adults [19,20]. Comparative cross-cultural and multi-generational trials remain a clear agenda item. Finally, our literature base was assembled through a purposive, narrative search: we prioritized important works in related human-automation trust, human–machine communication, and human–computer interaction literature to ground the CCC constructs; and searched for illustrative cases in sensors and related technology literature, rather than conducting a formal systematic review. This strategy risks overlooking less-visible empirical papers, future systematic reviews could therefore refine the framework’s applicability for different sensor use-cases.

Looking forward, technological evolution poses an external validity risk. Rapid advances in technology can reshape current stereotypes about AI accuracy and empathy. Ongoing validation studies should therefore treat CCC as an evolving hypothesis set, revisiting each construct as users’ mental models and regulatory landscapes mature. Acknowledging these boundaries helps prevent over-extension: CCC is a conceptual framework, not a universal law—and its greatest value lies in guiding targeted design and implementation interventions.

This article is deliberately conceptual, but CCC is readily testable. A phased program can convert the framework into cumulative evidence. (1) Content validity: map each construct to existing instruments (e.g., Machine-Heuristic, Perfect-Automation Schema) and develop new items for demonstrability and contrast. (2) Construct validity: run controlled-lab studies that orthogonally manipulate task demonstrability (high vs. low) and replacement mode (augment vs. displace) while logging behavioral trust, override frequency, and error-tolerance (e.g., [51]). (3) External validity: deploy CCC measures in diverse, real-world sensor settings such as consumer wearables, industrial safety monitors, clinical decision aids; and track trust trajectories over time to see whether the same pathways operate across contexts. Finally, (4), cross-cultural survey panels using the Machine-Heuristic and Perfect-Automation Schema items can model how baseline Cognition stereotypes differ by age and culture. Together, these steps provide a roadmap for moving the framework from conceptual to empirically grounded across low- and high-stakes AI biosensor deployments.

## 8. Conclusions

This paper reframes user trust in AI-enabled biomedical sensors through a Cognition–Context–Contrast lens. The model clarifies why considering user expectations, user education, context-aware communication, and carefully planned roll-outs are core design considerations. By formally uniting context, cognition, and contrast, CCC fuses three constructs that have never been combined in biosensor trust research. In turn, it closes conceptual gaps left by prior theoretical work and it does so at a time when the newest AI sensor innovations are reaching patients and clinicians who demand accuracy and confidence.

First, we outlined some of the fundamental expectations humans have about machines and artificial intelligence. Second, we showed that many trust hurdles become predictable once a sensor is placed on the intellective-versus-judgmental spectrum. Third, we sketched how each CCC dimension can inform broad design choices—such as providing transparent cues about how an algorithm reached its decision, matching the communication style to the certainty of the measurement, and introducing new systems alongside existing practice before a full hand-over.

These findings meet the study’s goals, weaving previously isolated insights from fields like human–automation trust and human factors into one coherent framework, showing how that framework can guide future research and development, and outline practical considerations that engineers, clinicians, and administrators can begin applying today. Taken together, we see the CCC framework as a conceptual lens and a pragmatic tool for researchers, designers, and organizational leaders to carry the next generation of AI wearables from proof-of-concept to standard of care.

## Data Availability

Data is contained within the article.
